# Implementation of and Barriers to Optimizing Postpartum Care by Resident and Attending Physicians

**DOI:** 10.31486/toj.22.0119

**Published:** 2023

**Authors:** Paige G. Pettus, Lucey W. Gavlinski, Shannon Beermann, Andrew G. Chapple, Stacey A. Scheib, Tabitha M. Quebedeaux, Adriene Louviere, Elizabeth F. Sutton, Stacey L. Holman

**Affiliations:** ^1^ Department of Obstetrics and Gynecology, Louisiana State University School of Medicine, New Orleans, LA; ^2^ Department of Interdisciplinary Oncology, Louisiana State University School of Medicine, New Orleans, LA; ^3^ Research Department, Woman's Hospital, Baton Rouge, LA

**Keywords:** *Delivery of health care*, *postpartum period*, *pregnancy*

## Abstract

**Background:** The American College of Obstetricians and Gynecologists released Committee Opinion No. 736: Optimizing Postpartum Care (CO No. 736) to address severe maternal morbidity and mortality in the United States by outlining recommendations for care in the critical time following birth. This study aimed to evaluate implementation of and barriers to the recommendations of CO No. 736 among obstetricians in south Louisiana.

**Methods:** A survey to general obstetric providers assessed opinions on the CO No. 736 recommendations, implementation of these recommendations, and barriers to implementation. Fisher exact test was used to compare distributions between resident and attending groups. Qualitative, free-text responses about barriers to implementation were organized by common themes and categorized into systemic and patient factors.

**Results:** Of 124 survey responses, 59.7% of respondents reported that they had read CO No. 736. Of the respondents who had read the document, 86.5% believed it was important to implement these recommendations, but only 50.0% had established the recommendations in their practices. Overall, fewer than half (46.8%) of respondents reported actively implementing the recommendation to make contact with postpartum patients at 3 weeks or sooner, but 86.3% reported having comprehensive clinic visits within 12 weeks of delivery. Commonly identified systemic barriers to implementation included the 3-week contact not being common practice, overbooked schedules, and unclear provider expectations. Commonly identified patient factor barriers to implementation included childcare or transportation and no-shows at postpartum appointments.

**Conclusion:** Both resident and attending obstetricians in South Louisiana believe that the CO No. 736 recommendations are important but reported lacking the ability to implement them into clinical practice.

## INTRODUCTION

In May 2018, the American College of Obstetricians and Gynecologists (ACOG) released Committee Opinion No. 736: Optimizing Postpartum Care (CO No. 736) to address severe maternal morbidity and mortality in the United States by outlining recommendations for care during the critical time following birth.^1^ The recommendations were a shift from the historic single clinical encounter to ongoing postpartum care that concentrates on a woman's individual needs. CO No. 736 contains 10 recommendations that focus on the continuum of care during pregnancy and the postpartum period and address the timing and content of provider assessments, reproductive life planning, and the management of medical conditions. With growing evidence for improved outcomes based on optimization of postpartum care,^2-5^ CO No. 736 was developed to support the implementation of these recommendations into clinical practice. The opinion, developed by the ACOG Presidential Task Force on Redefining the Postpartum Visit and the Committee on Obstetric Practice,^1^ has been endorsed by key stakeholders in the field of maternal health.

The goal of this study was to understand postpartum care practices in south Louisiana, a state with maternal mortality rates higher than the national average.^6^ We purposely focused on practices caring for an underserved population at risk for higher rates of maternal morbidity and mortality. The primary aim of our study was to survey if obstetricians in south Louisiana are applying or attempting to apply the CO No. 736 recommendations in their office practices. A secondary aim of our study was to identify perceived barriers to implementation of the CO No. 736 recommendations. We were specifically interested in the frequency of postpartum follow-up among providers, considering that CO No. 736 recommends a patient encounter within 3 weeks of delivery. We posited that the majority of obstetrics and gynecology (OB/GYN) providers surveyed were not implementing contact within 3 weeks postpartum but were implementing the recommendation for a comprehensive visit within 12 weeks.

## METHODS

The study team disseminated a 17-item survey (Figure) in person and electronically to OB/GYN providers, both resident (postgraduate year [PGY]-1 through PGY-4) and attending physicians. Nurse practitioners and nurse midwives were excluded. Providers were employed by academic medical centers (n=3) and private practices (n=7) in the greater New Orleans and Baton Rouge areas in Louisiana. The study was approved and monitored by the Louisiana State University Health Sciences Center (FWA00002762) and Woman's Hospital Foundation (FWA00005699) institutional review boards, and informed consent was obtained prior to survey initiation.

**Figure.**
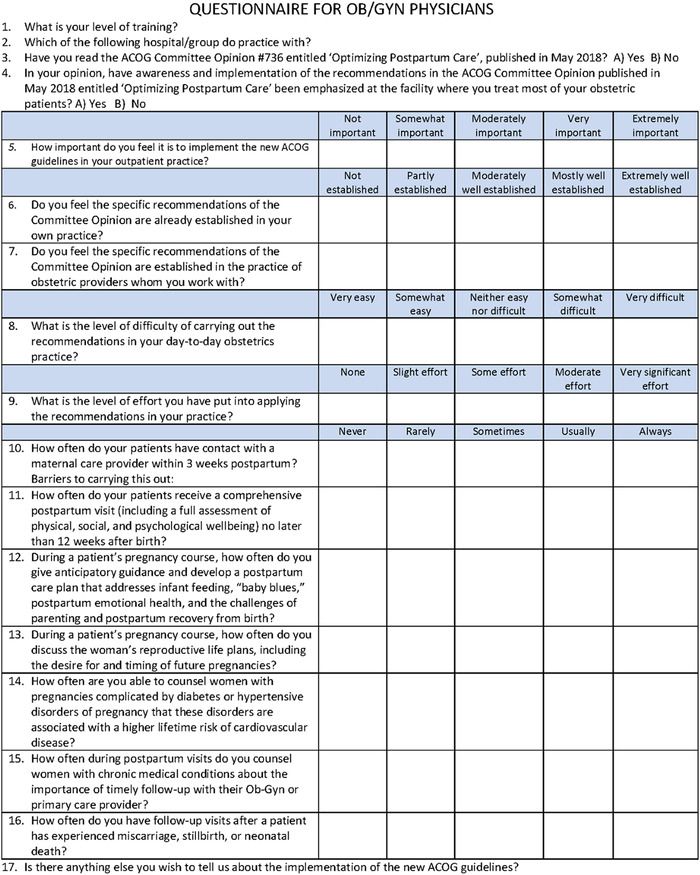
**Study survey.** ACOG, American College of Obstetricians and Gynecologists; OB/GYN, obstetrics and gynecology.

Surveys were anonymous, and no identifying information was collected from respondents. In New Orleans, surveying took place from June through September 2019 and was conducted in person at OB/GYN Grand Rounds, department meetings, and resident didactics sessions. In Baton Rouge, surveying took place from October through November 2020. Surveying was conducted in person in the physicians’ lounge, but the majority of the surveys at all sites were collected electronically using a REDCap (Vanderbilt University) survey link. Study data collected on paper were transcribed to REDCap for data aggregation. Study data were collected and managed using REDCap electronic data capture tools.^7,8^ REDCap is a secure, web-based software platform designed to support data capture for research studies, providing (1) an intuitive interface for validated data capture; (2) audit trails for tracking data manipulation and export procedures; (3) automated export procedures for data downloads to common statistical packages; and (4) procedures for data integration and interoperability with external sources.

The 17 questions on the survey (Figure) were informed by recommendations from CO No. 736 and were developed by the investigative team.^1^ Level of training, location of practice, and knowledge of CO No. 736 were assessed in survey questions 1 through 3. All survey participants answered 11 questions (questions 1 through 3 and 10 through 17), but only participants who responded “yes” to having previously read CO No. 736 answered 6 of the questions (questions 4 through 9). Responses were 5-point Likert scales. A blank space after each Likert scale question was provided to allow respondents to voluntarily describe barriers that they faced in implementing that specific recommendation. Question 17 invited free-text responses regarding implementation of the recommendations.

Responses were grouped into 2 categories: low implementation of recommendations (“never,” “rarely,” or “sometimes” responses) and high implementation of recommendations (“usually” or “always” responses). To compare the distribution of answers among resident and attending physicians, Fisher exact test was used to compare the resident and attending physician groups to test differences in training level on implementation of recommendations. This approach avoided the low response counts if Likert score values were tested individually, which would cause the test to behave poorly. *P* values <0.05 were considered statistically significant.

Researchers coded qualitative responses based on common themes that fell into 2 categories—systemic factors and patient factors—using a general inductive approach after all results were collected. These qualitative responses are also presented by training level. Because our study was exploratory in nature and without a specific hypothesis to test or preliminary data, a power analysis was not conducted.

## RESULTS

### Survey Respondents

A total of 124 surveys were collected from resident (n=44) and attending (n=80) OB/GYN providers from an available physician pool of 225, for an overall response rate of 55.1%. The response rates for New Orleans physicians were 52.9% (n=68) and 46.2% (n=78) for residents and attendings, respectively. The response rates for Baton Rouge physicians were 50.0% (n=16) for residents and 69.8% (n=63) for attendings, respectively.

### Compliance With Postpartum Care Recommendations

In addition to respondent training level and geographic location, Table 1 presents the frequencies of self-reported responses regarding implementation of the postpartum care recommendations in the respondents’ practices. Only 59.7% of respondents reported that they had read CO No. 736. Overall, respondents reported high implementation of specific recommendations of CO No. 736, with the “usually”/“always” percentages ranging from 46.8% to 98.4%. Attendings consistently reported higher compliance with the implementation of recommendations compared to residents.

**Table 1.** Survey Respondent Characteristics and Frequency of Implementation of American College of Obstetricians and Gynecologists Committee Opinion No. 736 Recommendations

Survey Question	All, n=124	Resident Physicians, n=44	Attending Physicians, n=80	*P* Value
**Respondent characteristic**				
Location				
New Orleans	72 (58.1)	36 (81.8)	36 (45.0)	
Baton Rouge	52 (41.9)	8 (18.2)	44 (55.0)	
Read Committee Opinion No. 736	74 (59.7)	27 (61.4)	47 (58.8)	0.85
**Postpartum care implementation**				
Question 10: How often do your patients have contact with a maternal care provider within 3 weeks postpartum?				
Usually/Always	58 (46.8)	15 (34.1)	43 (53.8)	0.04
Never/Rarely/Sometimes	66 (53.2)	29 (65.9)	37 (46.3)	
Question 11: How often do your patients receive a comprehensive postpartum visit (including a full assessment of physical, social, and psychological wellbeing) no later than 12 weeks after birth?				
Usually/Always	107 (86.3)	32 (72.7)	75 (93.8)	0.002
Never/Rarely/Sometimes	17 (13.7)	12 (27.3)	5 (6.3)	
Question 12: During a patient's pregnancy course, how often do you give anticipatory guidance and develop a postpartum care plan that addresses infant feeding, “baby blues,” postpartum emotional health, and the challenges of parenting and postpartum recovery from birth?				
Usually/Always	88 (71.0)	25 (56.8)	63 (78.8)	0.013
Never/Rarely/Sometimes	36 (29.0)	19 (43.2)	17 (21.3)	
Question 13: During a patient's pregnancy course, how often do you discuss the woman's reproductive life plans, including the desire for and timing of future pregnancies?				
Usually/Always	107 (86.3)	35 (79.5)	72 (90.0)	0.171
Never/Rarely/Sometimes	17 (13.7)	9 (20.5)	8 (10.0)	
Question 14: How often are you able to counsel women with pregnancies complicated by diabetes or hypertensive disorders of pregnancy that these disorders are associated with a higher lifetime risk of cardiovascular disease?				
Usually/Always	107 (87.7)	36 (85.7)	71 (88.9)	0.773
Never/Rarely/Sometimes	15 (12.3)	6 (14.3)	9 (11.3)	
Question 15: How often during postpartum visits do you counsel women with chronic medical conditions about the importance of timely follow-up with their Ob-Gyn or primary care provider?				
Usually/Always	122 (98.4)	43 (97.7)	79 (98.8)	1
Never/Rarely/Sometimes	2 (1.6)	1 (2.3)	1 (1.3)	
Question 16: How often do you have follow-up visits after a patient has experienced miscarriage, stillbirth, or neonatal death?				
Usually/Always	118 (95.9)	39 (90.7)	79 (98.8)	0.05
Never/Rarely/Sometimes	5 (4.1)	4 (9.3)	1 (1.3)	

Note: Data are reported as n (%).

A composite score (0-7) was calculated for each survey respondent based on their self-reported high or low implementation of each of the 7 primary recommendations of CO No. 736: contact within 3 weeks of giving birth; comprehensive postpartum visit no later than 12 weeks after birth; discussion of postpartum care plan; discussion of reproductive life plans; counseling about future cardiovascular disease risk; counseling for chronic medical conditions; and follow-up visit after miscarriage, stillbirth, or neonatal death. The mean composite score for attending OB/GYN providers was 6.0 ± 1.1, which was slightly higher than the OB/GYN resident mean score of 5.2 ± 1.2 (*P*=0.001). Both groups reported high implementation levels for the majority of the recommendations.

The lowest compliance rate was for the recommendation that patients have contact with a maternal care provider within 3 weeks postpartum. Only 34.1% of OB/GYN residents reported “usually”/“always” having contact with patients within 3 weeks of delivery vs 53.8% of OB/GYN attendings, a statistically significant difference (*P*=0.04). Both residents (72.7%) and attendings (93.8%) reported high implementation of the recommendation for a comprehensive postpartum visit within 12 weeks following delivery; however, implementation among attendings was significantly higher than among residents (*P*=0.002).

The recommendation with the second lowest implementation rate was providing anticipatory guidance and developing a postpartum care plan that addresses infant feeding, “baby blues,” postpartum emotional health, and the challenges of parenting and postpartum recovery from birth. Overall, 71.0% of respondents reported high implementation of this recommendation, with a significantly lower implementation reported by residents (56.8%) vs attendings (78.8%) (*P*=0.013). For the recommendations to discuss reproductive life plans and to counsel patients with pregnancies complicated by diabetes or hypertensive disorders that these disorders are associated with a higher lifetime risk of cardiovascular diseases, high implementation was reported overall by 86.3% and 87.7% of respondents, respectively, with no differences seen between residents and attendings in the reported rates of implementation.

The 2 recommendations for which respondents reported the greatest level of high implementation were counseling patients with chronic medical conditions about the importance of timely follow-up (98.4% overall; high implementation reported by 97.7% of resident and 98.8% of attending respondents, with no difference between residents vs attendings; *P*=1) and follow-up visits after a miscarriage, stillbirth, or neonatal death (95.9% overall; high implementation reported by 90.7% of resident and 98.8% of attending respondents, with a marginal difference between residents vs attendings but not significant; *P*=0.05).

### Opinions About Postpartum Care Recommendations

Survey respondents who reported that they had read CO No. 736 (n=74) were asked additional questions about implementing the recommendations in their clinical practice (Table 2). Overall, respondents reported poor awareness and implementation of the CO No. 736 recommendations in their facility, with only 27.0% reporting that they felt awareness and implementation had been emphasized where they practice. While 86.5% of respondents reported feeling the recommendations were “very important” or “extremely important,” 36.5% of respondents reported a high level of difficulty (“somewhat difficult” and “very difficult”) associated with carrying out the recommendation in their day-to-day obstetric practice. None of the resident and attending physician responses to these questions were significantly different.

**Table 2.** Survey Responses From Readers of American College of Obstetricians and Gynecologists (ACOG) Committee Opinion No. 736

Survey Question	All, n=74	Resident Physicians, n=27	Attending Physicians, n=47	*P* Value
Question 4: In your opinion, have awareness and implementation of the recommendations in the ACOG Committee Opinion published in May 2018 entitled ‘Optimizing Postpartum Care’ been emphasized at the facility where you treat most of your obstetric patients?				
Yes	20 (27.0)	7 (25.9)	13 (27.7)	1
No	54 (73.0)	20 (74.1)	34 (72.3)	
Question 5: How important do you feel it is to implement the new ACOG guidelines in your outpatient practice?				
Very/Extremely important	64 (86.5)	24 (88.9)	40 (85.1)	0.738
Not/Somewhat/Moderately important	10 (13.5)	3 (11.1)	7 (14.9)	
Question 6: Do you feel the specific recommendations of the Committee Opinion are already established in your own practice?				
Mostly/Extremely well established	37 (50.0)	9 (33.3)	28 (59.6)	0.052
Not/Partly/Moderately well established	37 (50.0)	18 (66.7)	19 (40.4)	
Question 7: Do you feel the specific recommendations of the Committee Opinion are established in the practice of obstetric providers whom you work with?				
Mostly/Extremely well established	31 (41.9)	13 (48.1)	18 (38.3)	0.468
Not/Partly/Moderately well established	43 (58.1)	14 (51.9)	29 (61.7)	
Question 8: What is the level of difficulty of carrying out the recommendations in your day-to-day obstetrics practice?				
Somewhat/Very difficult	27 (36.5)	13 (48.1)	14 (29.8)	0.137
Very easy/Somewhat easy/Neither easy nor difficult	47 (63.5)	14 (51.9)	33 (70.2)	
Question 9: What is the level of effort you have put into applying the recommendations in your practice?				
Moderate/Very significant effort	42 (56.8)	14 (51.9)	28 (59.6)	0.627
None/Slight effort/Some effort	32 (43.2)	13 (48.1)	19 (40.4)	

Note: Data are presented as n (%).

### Barriers to Postpartum Care Recommendations

Free-text qualitative data were collected for respondents who answered anything except a score of 5 (“always”) on the 5-point Likert scale for questions 10 through 16. Comments about the barriers to contacting patients within 3 weeks of delivery (the recommendation with the lowest implementation) were organized by theme—systemic factors or patient factors—and reported as resident and attending physician responses (Table 3). Both residents and attendings reported that major systemic barriers to implementation of the recommendation were that contact within 3 weeks postpartum was not routine practice and that schedules were overbooked. Residents also commented that they were unclear whose role it should be to schedule and provide contact with patients within 3 weeks postpartum, with some respondents commenting that midlevel providers (ie, nurse practitioners and nurse midwives) might be more appropriate for performing this role than physicians. Patient factor barriers included the difficulty of arranging childcare or transportation and appointment no-shows.

**Table 3.** Barriers to Implementation of Postpartum Contact by 3 Weeks

Theme	Barrier	Resident Physician Comments, n=24	Attending Physician Comments, n=38
Systemic factors	Not common practice	6	6
	Overbooked schedules	7	9
	Unclear whose role it is/use of midlevels	4	1
	Insurance coverage/reimbursement	2	5
Patient factors	Childcare or transportation	8	10
	Support/psychological	1	3
	No-show	3	9
	Patient understanding/perception	2	0

Comments for barriers to implementing a comprehensive postpartum visit by 12 weeks were also organized by theme—systemic factors or patient factors—and reported as resident and attending physician responses (Table 4). Mirroring the comments regarding the patient factor barriers to the 3-week contact, both residents and attendings felt that patients’ difficulty obtaining childcare or transportation and patient no-shows were barriers to implementing the visit by 12 weeks.

**Table 4.** Barriers to Implementation of Comprehensive Postpartum Visit by 12 Weeks

Theme	Barrier	Resident Physician Comments, n=22	Attending Physician Comments, n=21
Systemic factors	Not common practice	0	0
	Overbooked schedules	3	1
	Unclear whose role it is/use of midlevels	1	0
	Insurance coverage/reimbursement	1	2
Patient factors	Childcare or transportation	4	5
	Support/psychological	0	2
	No-show	13	15
	Patient understanding/perception	0	0

For both the 3-week contact and the comprehensive visit by 12 weeks, attendings cited problems with insurance coverage for patients and reimbursement for physicians as barriers to implementation more frequently than residents.

## DISCUSSION

Among the surveyed population representing a diverse snapshot of OB/GYN providers in the Baton Rouge and New Orleans areas, our survey study revealed that only 59.7% of the respondents had read the ACOG Committee Opinion No. 736: Optimizing Postpartum Care. Regardless of whether respondents had or had not read CO No. 736, our study revealed that the majority of respondents were implementing the opinion recommendations to a limited degree. We found that the postpartum care recommendations were implemented less frequently among residents compared to attending OB/GYN providers.

Our study provides important insight from OB/GYN providers into feelings about and barriers to implementation of the ACOG postpartum care optimization recommendations. Implementation of these recommendations deserves thoughtful consideration as more than half of pregnancy-related deaths occur after delivery.^9^ Consequently, the postpartum period must be part of any strategy to reduce maternal morbidity and mortality. Particularly relevant to the surveyed population, Louisiana has one of the highest maternal mortality rates in the country, and the mortality rate is increasing at a higher rate than the rest of the United States.^9^ Postpartum care practices contribute to maternal morbidity and mortality,^1^ making this phase of care an important topic for all providers. Credit should be given to CO No. 736 for recommending postpartum care as an ongoing process rather than an isolated visit at 6 to 12 weeks after delivery. Ultimately, full implementation of these recommendations to change the scope of postpartum care must be facilitated by reimbursement policy.

Free-text responses provided insight into perceived barriers that may be limiting implementation of the CO No. 736 recommendations, and both resident and attending OB/GYN providers provided similar insights. The most commonly reported barriers to implementation of making contact by 3 weeks postpartum and completing a comprehensive visit by 12 weeks were consistent among residents and attendings and included overbooked schedules, unclear roles of providers for these visits, patient no-shows, and patient transportation or childcare. Respondent suggestions for alleviating these barriers included using midlevel providers to schedule appointments, contacting patients during the 3-week postpartum window, and assisting with patient reminders for postpartum visits.

Moreover, those who had read CO No. 736 reported feeling that their facility had not implemented the recommendations despite their opinion that the recommendations are important. Institutional support, both endorsement and allocation of resources, for piloting the 3-week postpartum contact is an opportunity to help providers implement this recommendation into their practices.

An additional common barrier to postpartum care recommendations that deserves discussion is the report of no-shows to postpartum appointments. Approximately 60% of patients attend a postpartum visit in the United States,^1^ with lower attendance rates for patient populations with limited resources.^10^ At the academic medical centers where the surveys for this study were disseminated, attendance rates for the comprehensive postpartum clinic visit 6 to 12 weeks after delivery were approximately 50% in Baton Rouge and 42% in New Orleans in 2021, both below the national average.

Incorporation of additional visits or contacts into the standard postpartum visit schedule would require considerable effort to support patient compliance as attendance at the standard 6- to 12-week postpartum visit is already lacking. However, increasing patient contact in the postpartum period stands as a potentially high-reward opportunity among the obstetric population who experience both limited postpartum follow-up and high rates of pregnancy-related morbidity in the postpartum period.

Our study has strengths, limitations, and opportunities for future work. A strength of our study is sampling among multiple academic centers (n=3) in 2 regions in south Louisiana. Another strength is receipt of physician responses across several different practice groups and settings, particularly among attending physicians in Baton Rouge and resident physicians in New Orleans. A limitation is the low response rate among all the eligible providers in the regions sampled. Another limitation is the inability to distinguish academic vs private practice responses because of the anonymity of the survey. Regarding future opportunities with this work, many barriers reported are organizational barriers; therefore, future studies should focus on investigating specific organizational opportunities to implementation of postpartum care. Expansion of this work will include statewide dissemination to obstetric providers to increase responses and gain insight outside of urban settings. Future retrospective studies could also be performed that use actual occurrence rates of postpartum care practices rather than provider self-reported rates. Suggestions to begin working toward the implementation of the optimal postpartum care recommendations include supporting obstetric practices in implementing the recommendations from CO No. 736, emphasizing postpartum care at prenatal visits, using support staff to remind patients of their appointments, using midlevel providers to assist with schedule burden, emphasizing postpartum visits in discharge planning, scheduling postpartum telehealth visits and comprehensive visits at discharge, and sending text or email reminders. Advocacy for support of statewide insurance coverage for the 3-week visit could also reduce barriers to implementing that visit. Providing childcare services in clinic and allowing children to accompany the patient for the first postpartum visit could also potentially assist in boosting attendance rates. Looking ahead, we are conducting a quality improvement project in which these insights will be applied to a 3-week postpartum telehealth visit with patients in an academic resident clinic.

## CONCLUSION

Our survey results show that the ACOG Committee Opinion No. 736: Optimizing Postpartum Care recommendations are being implemented intermittently by both resident and attending OB/GYN providers in South Louisiana. The barriers are several and vary depending on the provider and training level. This work should serve as a reminder of the importance of postpartum comprehensive care in reducing maternal morbidity and mortality in the state of Louisiana. We also hope that the findings from this study serve as a platform to advocate for the reduction of financial barriers across the state to allow patients to access this recommended model of care.

## Notes

*This article meets the Accreditation Council for Graduate Medical Education and the American Board of Medical Specialties Maintenance of Certification competencies for Patient Care and Medical Knowledge.*
